# Visual imagery ability in the continuum of Alzheimer’s disease: the mind’s eye compared in patients with MCI and early AD

**DOI:** 10.1002/alz.092311

**Published:** 2025-01-03

**Authors:** Giulia Bechi Gabrielli, Roberta Margiotta, Massimiliano Panigutti, Fabrizia D'Antonio, Antonella Di Vita, Maddalena Boccia, Liana Palermo, Laura Piccardi, Micaela Sepe‐Monti, Giuseppina Talarico, Emanuela Salati, Giuseppe Bruno, Cecilia Guariglia

**Affiliations:** ^1^ Sapienza University of Rome, Rome, Rome Italy; ^2^ Magna Graecia University, Rome, Rome Italy

## Abstract

**Background:**

Visual Mental Imagery (VMI) is the ability to represent stimuli in the mind without sensory visual input. Previous studies have shown alterations in visual imagery in Alzheimer’s disease (AD). However, VMI has not been investigated in the AD prodromal stage, i.e., the Mild Cognitive Impairment (MCI). In this study, we aimed to investigate the VMI ability in AD and MCI patients. We hypothesized that VMI ability could be compromised from the early disease stage.

**Method:**

For our purpose, we enrolled 14 patients with a diagnosis of probable typical AD, 19 amnestic MCI (aMCI), and 23 healthy control subjects (HC), matched for sex, age, and education; MMSE scores in AD patients significantly differed from those in aMCI and HC, that not differed between them. The extensive assessment of VMI included: 1) the O’Clock test that allows disentangling the possible role of visuo‐perceptual difficulties in the VMI task’s performance; 2) a modified version of The Complete Visual Mental Imagery Battery (CVMIB), including tasks evaluating the different VMI processes (Kosslyn, 1980; 2005), that is generation, maintenance, inspection and transformation of different types of visual mental images (i.e., objects, buildings and faces).

**Result:**

Results indicated that AD patients performed worse than HC in both perceptual and imaginal tasks of the O’Clock test and in all CVMIB’s tasks but maintenance. On the contrary, aMCI patients showed difficulties in the generation process and the imaginal task of the clock test.

**Conclusion:**

Our findings suggested that generation, inspection and transformation processes of VMI are impaired since the early phase of Alzheimer’s disease. Moreover, we found that the generation process is selectively impaired in aMCI patients, demonstrating that VMI deficits are already present in the prodromal stage of the disease. Since alterations in VMI processing might reflect the AD neuropathological progression, according to Braak and Braak’s model, VMI abilities might represent a useful neuropsychological marker of dementia to identify AD prodromal stage.

